# Needs Assessment to Inform a Life Course-Based Support Programme for Adolescents with Perinatally Acquired HIV in Rural Limpopo, South Africa

**DOI:** 10.3390/ijerph23040441

**Published:** 2026-03-31

**Authors:** Rirhandzu Austice Mabasa, Cairo Bruce Ntimana, Tebogo Maria Mothiba, Linda Skaal

**Affiliations:** 1 Department of Public Health, University of Limpopo, Sovenga St., Polokwane 0727, South Africa; 2 DIMAMO Population Health Research Centre, University of Limpopo, Sovenga St., Polokwane 0727, South Africa; cairo.ntimane@ul.ac.za; 3 Faculty of Health Sciences, Executive Deans Office, University of Limpopo, Polokwane 0700, South Africa; tebogo.mothiba@ul.ac.za; 4 Department of Public Health, Faculty of Health Sciences, Sefako Makgato University, Ga-Rankuwa 0208, South Africa; linda.skaal@smu.ac.za

**Keywords:** perinatal HIV, life course, theory, adolescents, rural

## Abstract

**Highlights:**

**Public health relevance—How does this work relate to a public health issue?**

- The community of adolescents with perinatal HIV represents a growing and vulnerable population facing poor ART adherence, mental health challenges, and increased risk of morbidity and onward transmission, particularly in rural South Africa.
- The Life Course in this study assisted in the development of a support programme to address structural, social, as well as other determinants of health that contribute to inequities in HIV outcomes in rural Limpopo.

**Public health significance—Why is this work of significance to public health?**

- The findings of this study pointed out that improving psychosocial support and sustained ART adherence during adolescence is critical for achieving viral suppression and reduction in HIV transmission.
- This work further contributes to preventing health system failure by preventing treatment failure and drug resistance, which might contribute to health system burden.

**Public health implications—What are the key implications or messages for practitioners, policy makers and/or researchers in public health?**

- Public health programmes should adopt life course-based, adolescent-centred models that integrate psychosocial care, family support, and transition planning into routine HIV services in rural settings.
- Policy makers and researchers should prioritize context-specific interventions for adolescents with perinatal HIV and generate evidence on scalable, community-based support models that strengthen continuity of care across the life span.

**Abstract:**

This study explored the challenges and support needs of adolescents identified as living with perinatally acquired HIV based on clinical history and early childhood treatment records to generate evidence that can inform the development of an adolescent-centred support programme. A mixed-methods, sequential exploratory design was employed. In the qualitative phase, semi-structured interviews were conducted with 21 APHIV (mean age 15.5 years, range 12–19; 61.9% female) recruited from health facilities in rural Limpopo, South Africa. In the quantitative phase, a survey informed by qualitative findings was administered to 186 APHIV. Life Course Theory informed the conceptual framework of this study and guided the development of the interview guide as well as the interpretation of adolescents’ lived experiences. Data saturation was reached after twenty-one in-depth interviews. The qualitative analysis generated themes related to HIV/AIDS knowledge and support systems, which were subsequently interpreted through key Life Course Theory constructs, including time and place, agency, timing, and linked lives. The findings revealed that the life trajectories of older adolescents were more strongly shaped by the historical circumstances surrounding their HIV diagnosis and disclosure compared with those of younger adolescents. The results further highlighted gaps in treatment literacy, challenges in disclosure processes, emotional distress, and variability in family and health-care support. Most adolescents reported that their HIV-positive status was disclosed by someone other than their parents, while some discovered their status independently. The findings provide empirical evidence on informational, psychosocial, and support needs that can guide the design of adolescent-centred interventions for adolescents living with perinatally acquired HIV. This study recommends the development of context-specific, adolescent-centred support programmes that integrate psychosocial support, family engagement, and improved communication with healthcare providers to strengthen adherence and well-being among APHI.

## 1. Introduction

Advanced HIV care and management afforded people living with HIV, including adolescents who were born with and are living with HIV [1]. Parallel to the transitioning challenges of the adolescent stage, adolescents living with perinatal HIV(APHIV) face unique and additional HIV-related challenges as they navigate the complexities of growing and living with HIV [2]. These challenges highlight the need for a focused and specific support programme to assist them in navigating the stages of life as informed individuals [2].

South Africa continues to rank high amongst high-burden countries with HIV/AIDS in African countries [3]. According to recent HIV statistics, an estimated 40.8 million people were living with HIV by the end of 2024 [4], with approximately 1.4 million children (aged 0–14 years) and millions more adolescents living with the infection globally [4]. Adolescence is a stage in human development, marked by significant physical, emotional, and social changes [5]. For adolescents identified as having perinatally acquired HIV, parallel to the long-term effects of living with HIV, are unique challenges associated with living with HIV from early childhood [6]. HIV medication and advanced HIV management and care improved life expectancy and the quality of life for individuals, including APHIV [7]. The developmental needs of perinatally HIV-infected adolescents remain underexplored and under-addressed [8]. Furthermore, HIV management guidelines have a comprehensive framework on the management of HIV positive patients or ART; however, they fail to recognize the unique needs of the APHIV population [9,10]. It provides an umbrella approach to all HIV positive adolescents, both horizontally and vertically infected [10]. Consequently, there is a significant loss to follow-up among adolescents and eventually absconding from HIV/AIDS care and management [11]. Furthermore, in South Africa, there is an absence of proper guidelines and a support system to help these patients transition safely throughout adolescence, often leading to poor decision making and high-risk behaviours among APHIV [12,13]. The alarming rate of risky behaviours amongst APHIV underscores the trajectories in transitioning to adulthood [14,15,16]. Studies report increasing rates of unprotected sex and unplanned pregnancies among APHIV, with pregnancies occurring as early as 10 years of age [17,18].

These gaps highlight the necessity for interventions that go beyond clinical management to include psychosocial, developmental, structural, and cultural dimensions. Understanding the lived experiences of APHIV requires a holistic approach. In this context, the Life Course Theory offers a useful framework, as it emphasizes how experiences across different life stages and generations shape health and behaviour [19]. The theory recognizes that individual development is influenced by interconnected biological, behavioural, and psychosocial pathways operating over time. Guided by this framework, the study developed an in-depth interview guide to explore APHIV experiences comprehensively.

Although several interventions exist to support adolescents living with HIV, there is limited context-specific evidence to guide the design of adolescent-centred support programmes for those with perinatally acquired HIV in rural South Africa [20,21]. Therefore, this study aimed to conduct a mixed-method needs assessment informed by Life Course Theory to better understand the lived experiences, informational needs, and support systems of adolescents with perinatal HIV. The findings are intended to inform the future development of a contextually relevant support programme.

## 2. Materials and Methods

### 2.1. Study Design

This study adopted a mixed-method, sequential exploratory design to explore the experiences and support needs of adolescents identified through clinical records as living with perinatally acquired HIV. The method allowed for exploring and identifying gaps in the lives of APHIV. This study, therefore, represents the exploratory needs-assessment phase intended to generate evidence for the future design of a Life Course-informed support programme.

### 2.2. Study Setting

This study was conducted in selected health facilities of the Vhembe district in Limpopo Province of South Africa. These facilities offer HIV care and management, which is run primarily by Nurse-Initiated and Managed Anti-Retroviral Treatment (NIMART). Three municipalities, which were Musina, Thulamela, and Collins Chabane, were selected to participate in this study.

### 2.3. Population and Sampling

The population comprised adolescents who were born with HIV and were taking ART at the selected facilities of the above-mentioned municipalities in the Vhembe District, Limpopo Province. A purposive sampling technique was applied to select 21 adolescents living with perinatal HIV who started antiretroviral therapy before the age of 10 years.

Inclusion criteria: Adolescents aged 10–19 years enrolled in HIV care at selected facilities. Documented evidence of perinatal HIV acquisition, confirmed through clinical records indicating: HIV diagnosis before age 5, or continuous enrolment in HIV care since early childhood (under age 5) with caregiver confirmation. Initiation of antiretroviral therapy before age 10 (as a proxy indicator consistent with perinatal infection). Knowledge of one’s own HIV status (to avoid unintentional disclosure during data collection).

Collecting ART independently for at least 6 months (to ensure relevance for transition-focused support needs.

Exclusion criteria: Adolescents who were diagnosed and started HIV care and management after the age of 10. Adolescents who were living with perinatally acquired HIV and were also living with a chronic mental illness that was not controlled, as their response could be influenced by their state of mind. APHIV were between the ages of 10 and 19, but were not collecting ARVs on their own from the health care facilities.

To confirm perinatal HIV acquisition, the research team reviewed clinic records and patient files in consultation with the attending nurses and lay counsellors who had long-term knowledge of the adolescents’ treatment histories. Perinatal HIV status was established based on documented evidence of: (1) HIV diagnosis in infancy or early childhood (under age 5) as recorded in the patient’s clinical file, (2) continuous enrolment in HIV care since early childhood, and (3) caregiver confirmation that the adolescent had been taking ART since before the age of 5. Participants with documented horizontal transmission (e.g., through sexual contact or blood transfusion) after age 5 were excluded. The age-10 cut-off for ART initiation was used as an additional proxy indicator, recognizing that children with perinatally acquired HIV would have necessarily initiated treatment well before adolescence, whereas those horizontally infected in later childhood would have a different treatment initiation trajectory.

### 2.4. Data Collection

The authors sought and requested permission to conduct identified facilities through operational managers as the gatekeepers of the facilities. APHIV was identified with the assistance of nurses, lay-counsellors, and data capturers who were working directly with them. For qualitative data collection, individual interviews were conducted with the assistance of research assistants using a semi-structured interview guide. Interviews lasted between 25 and 60 min per session. Interviews were conducted using the language of the participant, in this case, either Xitsonga or Tshivenda. Data was collected until saturation. Saturation was assessed using a codebook stability approach, where the research team monitored the emergence of new codes during iterative data analysis. After each interview, transcripts were reviewed, and new codes were documented. It was determined that saturation had been reached when three consecutive interviews yielded no new codes or themes, and when all a priori constructs from the Life Course Theory framework had been fully explored and exemplified. This occurred after twenty-one (21) one-on-one interviews, at which point the research team agreed that the data were sufficiently rich and comprehensive to address the research objectives. Back-to-back translation of the interview guide was done. It was prepared in English and translated into Xitsonga and Tshivenda, respectively, and back to English. The translation was done to allow all participants to express themselves in their own languages. It consisted of a series of preset questions, open-ended questions, supplemented by follow-up questions, clarification, probing, and comments.

For the quantitative strand, the developed questionnaire for adolescents living with perinatal HIV infection was informed by the findings of the themes and sub-themes of qualitative data collected. The questionnaire was sent to the supervisors for validation. The questionnaire consisted of 3 sections: section A: Demographic information of the participants, section B: Assessment of the challenges faced by adolescents living with perinatally acquired HIV, and section C: Assessment of the coping strategies of adolescents living with perinatally acquired HIV.

A total of 213 questionnaires were distributed, and of these, 186 were suitable for analysis. Questionnaires were hand-delivered to participants at locations chosen by participants for completion and collection. To protect confidentiality and minimize response bias, the following procedures were implemented:

Privacy safeguards: Participants were offered the choice of completing the questionnaire in a private space at the health facility, at home, or at another location of their preference. Where questionnaires were completed at health facilities, a private room was used with only the participant and, if requested, a researcher or research assistant present. For participants choosing home completion, they were instructed to complete the questionnaire in a private space away from family members and were provided with a sealed envelope to return the completed questionnaire.

Administration procedures: Questionnaires were self-administered by adolescents. For younger participants (ages 10–12) or those with limited literacy who required assistance (n = 14; 7.5% of the final sample), assistance was provided exclusively by a trained research assistant of the same gender as the participant, rather than a guardian, to reduce the risk of social desirability bias and ensure confidentiality of responses. The research assistant read questions aloud verbatim and recorded the participant’s exact responses without interpretation or prompting. Guardians were not present during questionnaire completion for any participant to prevent their influence on responses.

Confidentiality assurance: All participants were assured that their responses would remain confidential and that no clinic staff, family members, or community members would have access to their individual questionnaires. Unique study identifiers, rather than names, were used on all questionnaires. Completed questionnaires were placed in sealed envelopes by participants themselves before being returned to the research team.

Response bias consideration: The research team acknowledges that despite these safeguards, the presence of a researcher or research assistant during questionnaire completion may have introduced some degree of social desirability bias, particularly for sensitive questions about emotional distress, family relationships, and adherence. This limitation is addressed further in the Discussion section.

A pilot study was done in Madimbo Clinic, as their clinic sample constituted almost 10% of the whole sample, which was 20 participants. Participants in that clinic did not form part of the main study. Piloting of the questionnaire ensured that ambiguous questions were rectified before the main study data collection sessions started.

### 2.5. Data Analysis and Data Integration

Data was collected until saturation. In this study, data saturation was reached after twenty-one (21) one-on-one interviews were conducted by the researcher. Qualitative data were analyzed using Tesch’s (Creswell et al., 2023) eight steps of thematic analysis [22]. The analysis was conducted by a team comprising the primary researcher (RAM) and an independent coder with expertise in qualitative HIV research. To ensure consistency and rigour, the following coding procedures were implemented: Step 1: The primary researcher (RAM) read through all transcripts multiple times to gain immersion in the data and jotted down initial impressions and ideas on the margins of each transcript.

Step 2: One transcript was selected for detailed line-by-line coding. The researcher identified meaningful units of text and assigned preliminary codes to capture the essence of participants’ experiences. Step 3: A preliminary codebook was developed by listing all emerging codes, grouping similar codes, and organizing them into broader categories. This codebook included code definitions, inclusion criteria, and exemplar quotes. Step 4: The preliminary codebook was shared with the independent coder, who independently applied it to a subset of three transcripts. Both coders then met to compare coding applications, discuss discrepancies, and refine code definitions. Disagreements were resolved through consensus discussion, and where consensus could not be reached, a third reviewer (CBN) was consulted to make a final decision. This iterative process resulted in a finalized codebook. Step 5: Using the finalized codebook, the primary researcher coded all remaining transcripts. New codes that emerged during this process were documented and, where appropriate, integrated into the codebook after team discussion. Step 6: Related codes were grouped into categories, and categories were further abstracted into overarching themes and sub-themes. This involved identifying patterns, relationships, and connections across the data.

Step 7: The primary researcher and independent coder met again to review the proposed themes and sub-themes. Each theme was examined for internal homogeneity (coherence of data within a theme) and external heterogeneity (clear distinctions between themes). Final themes were agreed upon through consensus. Step 8: Themes and sub-themes were defined, named, and summarized. Illustrative quotations were selected to represent each theme, ensuring representation across age groups, gender, and geographic locations.

The analysis followed a distinct two-step process to ensure transparency in how findings were derived and interpreted. In the first step, an inductive thematic analysis was conducted using Tesch’s approach, allowing themes to emerge directly from the participants’ narratives without imposing theoretical preconceptions. This step generated the core qualitative themes presented in Table 4 (e.g., HIV/AIDS Knowledge, Support Systems). In the second step, these inductive themes were re-examined and interpreted through the lens of Life Course Theory (LCT). This involved mapping each emergent theme and sub-theme to relevant LCT constructs, such as ‘timing in lives,’ ‘linked lives,’ and ‘human agency, to understand the developmental and social mechanisms shaping adolescents’ experiences. This two-step approach explicitly distinguishes between what participants reported (inductive findings) and how these findings can be understood within a broader theoretical framework (theoretical interpretation).

Quantitative data were analyzed using Statistical Package for the Social Sciences (SPSS) Version 25 with the assistance of the university biostatistician. Data were organized in a spreadsheet and cleaned by removing duplicate entries and incomplete responses. Prior to analysis, all quantitative data were screened for completeness using the following criteria:

Missing data handling: Cases with extensive missing data, defined as more than 20% of the questionnaire incomplete across all sections, were excluded from the final dataset. This accounted for the reduction from 213 distributed questionnaires to 186 suitable for analysis (12.7% exclusion rate). For specific items and scales, missing data were handled using pairwise exclusion, meaning that for each analysis, only cases with complete data for that particular item were included. This approach explains the variation in sample sizes reported across tables (e.g., n = 170 for some emotional assessment items in Table 2, n = 186 for demographic items in Table 1). The denominator for each percentage is clearly indicated in table notes as the number of valid responses for that specific item. This approach maximizes the use of available data while maintaining transparency about response rates for individual questions.

Denominator reporting: Throughout all tables, the sample size (n) for each item was explicitly reported, and percentages were calculated based on the number of valid responses for that item rather than the total study sample, unless otherwise specified.

Qualitative and quantitative data were integrated or linked at the design level by using an exploratory sequential design. The questionnaire for quantitative data collection was designed based on the results of the qualitative data. Interpretation level—integration has been utilized to fully address the research questions connecting qualitative data from Phase 1 of this study and quantitative data from Phase 2. The qualitative interview results and statistical analysis results were compared, and areas of convergence were discussed to develop an overall understanding through integration of both data strands.

### 2.6. Integration of Qualitative and Quantitative Phases

This study employed an exploratory sequential design, where the qualitative phase (Phase 1) was conducted first to explore the lived experiences of APHIV, and its findings were used to build the quantitative phase (Phase 2). The integration occurred at two key levels: the design level and the interpretation level.

At the design level (instrument building): The findings from Phase 1, specifically the emergent themes and sub-themes (e.g., HIV/AIDS knowledge gaps, experiences of disclosure, types of support), served as the primary framework for developing the quantitative questionnaire. A “theme-to-item” matrix was created to ensure content validity. For each qualitative sub-theme, the research team drafted multiple potential questionnaire items. For instance, the sub-theme “*Knowledge of ARVs and related side effects*” directly informed the creation of items like “*Feel like I am not coping with taking medication every day*” and “*My lifestyle has changed since I started taking my ARVs.*” Similarly, the sub-theme “*Support from clinic nurses*” was translated into items such as “*The health professional attends to me when I come for a consultation*” and “*Wish nurses can also give attention when I come for my consultation.*” The initial pool of items was reviewed by three public health experts for face and content validity. The questionnaire was then refined based on feedback from the pilot study (n = 20), where ambiguous items were identified and reworded to ensure clarity and relevance for the target population.

At the interpretation level (narrative weaving and joint display), Integration was achieved by comparing and contrasting the qualitative findings with the quantitative results to develop a holistic understanding. This process, known as generating “meta-inferences,” is presented in the Discussion Section, where qualitative quotes and themes are discussed alongside quantitative percentages to provide a richer, more comprehensive interpretation of the challenges faced by APHIV. To further illustrate this integration, a Joint Display table (Table S1) has been created, which maps the core qualitative themes to their corresponding quantitative items and summarizes the integrated findings.

### 2.7. Ethical Approval and Informed Consent Statement

Permission was obtained from the University of Limpopo ethics committee (TREC/228/2019: PG, approved 4 September 2019), the Limpopo Department of Health, and health facility managers. This study was conducted in accordance with the Declaration of Helsinki. Only adolescents who knew their HIV status were included to avoid unintentional disclosure. Written informed consent was obtained from parents/guardians and adolescents aged 18 years and older; verbal assent was obtained from younger participants. Participants were informed of their right to withdraw at any time without penalty. The information sheet outlined the limits of confidentiality, including the obligation to break confidentiality if a participant was at risk of harm to self or others.

A safety protocol was implemented due to the sensitive nature of questions assessing emotional distress and suicidal ideation. All data collection sessions were conducted in the presence of a qualified professional nurse trained to recognize and respond to psychological distress. A two-tiered referral system was established: for mild distress, the session was paused and on-site counselling provided; for severe distress or endorsed suicidal ideation, the nurse conducted an immediate risk assessment and facilitated referral to a mental health counsellor, psychologist, or emergency services. All participants received referral cards with contact details for free counselling services and crisis support. A confidential log of all referrals was maintained using unique study identifiers.

### 2.8. Trustworthiness and Rigour

Trustworthiness was established using Lincoln and Guba’s four criteria. Credibility was achieved through prolonged engagement in the study setting, member checking with five participants to verify interpretations, triangulation of diverse data sources (age, gender, location) and methods (qualitative findings compared with quantitative survey data), and peer debriefing with the research team. Dependability was ensured through a comprehensive audit trail including raw transcripts, field notes, codebooks, and analytical memos, with an independent coder reviewing the coding process. Confirmability was addressed through reflexive journaling by the primary researcher to document personal biases, a transparent audit trail of all analytical decisions, and consensus-based coding by multiple researchers to minimize individual bias. Transferability was enhanced through thick description of the study context (rural Limpopo, Vhembe District), participant characteristics, and health facility settings, supported by rich verbatim quotations enabling readers to assess applicability to other contexts.

## 3. Results

This study included 186 adolescents identified as living with perinatally acquired HIV based on clinical records and early childhood treatment history. Slightly more participants were aged 16–19 years (51.1%) than 12–15 years (48.9%). Females made up 53.8% of the sample. Most participants had secondary education (62.4%), and the majority lived in rural areas (57%). More than half were staying with their parents (56.5%). About one-third (29%) had lost a parent due to HIV infection (Table 1).

Table 2 shows the emotional status of adolescents. Below a quarter of 29 (17.1%) of adolescents have had problems with other family members due to their positive HIV status. Also, close to a quarter of 40 adolescents (23.5%) experienced challenges with their schooling due to HIV infection. Approximately one-third of adolescents (56; 32.9%) reported feeling guilty about their HIV-positive status.

Most participants reported a need for more HIV/AIDS knowledge (54.8%), indicating ongoing educational gaps. While the majority did not feel helpless about their HIV status, a considerable proportion experienced psychological distress, including fear about the future, difficulty coping with daily medication, and feelings of losing control. Many participants expressed dissatisfaction with healthcare interactions, wishing for more attention, communication, and counselling from nurses. Family support was mixed, with some participants reporting supportive environments while others felt treated differently at home (Table 3).

The qualitative findings are presented in two integrated layers to maintain analytical clarity. First, inductive thematic analysis generated two overarching themes—HIV/AIDS Knowledge and Support Systems—with their respective sub-themes, representing the authentic voices and lived experiences of participants. Second, these inductive themes were subsequently interpreted through key constructs of Life Course Theory, including timing in lives, linked lives, human agency, and life-span development. Table 4 presents this integrated analytic structure, showing the inductive themes alongside their theoretical interpretation and supported by verbatim quotations with participant identifiers.

### 3.1. Theme 1: HIV/AIDS Knowledge

#### 3.1.1. Sub-Theme 1.1: Knowledge of HIV/AIDS and Self-HIV-Positive Status

Most adolescents demonstrated knowledge of HIV transmission, prevention, and what HIV is. Those in the 16–19 age group and in higher secondary school grades showed better understanding. All adolescents knew their HIV status, although many, especially those younger than 12, were reluctant to reveal it because of stigma.

“*HIV is a disease that is caused by mixing of two types of blood where one person is infected by HI virus… It can also be transmitted from the mother to an unborn child and when people have sexual intercourse without protection.*”

(Participant 002)

“*HIV is a virus which lives in a human body but when you take your medication it can be controlled.*”

(Participant 004)

“*I was told it is a disease which is transmitted through unprotected sexual intercourse, and it is deadly… a person will not die if he or she takes medication and follows instructions.*”

(Participant 006)

“*I don’t know anything about HIV, but I have heard that when I sleep with a man, I must use condoms and that I must take my pills every day.*”

(Participant 009)

“*Yes… I know that I am HIV positive.*”

(Participant 002)

“*I know that when you are HIV positive, you should not touch other people’s blood and that you must not play in dirty water.*”

(Participant 021)

“*HIV is that disease (vuvabyi lebyiya), it is not like any other disease.*”

(Participant 005)

“*I just don’t want to have HIV; I would rather take my medication without knowing what it is for…*”

(Participant 008)

“*This sickness of HIV that I have, I only heard people talking, saying ‘HIV, HIV’.*”

(Participant 001)

#### 3.1.2. Sub-Theme 1.2: Knowledge of ARVs and Related Side Effects

Most adolescents did not know the specific purpose of their medication, only that they had to take it daily. Some took treatment from a young age without understanding its purpose. Others reported that they relied on their parents’ guidance and did not question it. Some adolescents reported not experiencing side effects, while others mentioned dizziness, feeling sick, or treatment fatigue. The following extracts are what the participants said:

“*I don’t know what the treatment is for… I must take them every day, or I will die.*”

(Participant 016)

“*My father told me that if I don’t take my pills, I will get sick and go to the hospital… they will give me an injection that will kill me.*”

(Participant 001)

“*I don’t know; I was only given medication with the instructions only.*”

(Participant 003)

“*HIV is a virus which lives in a human body but when you take your medication it can be controlled.*”

(Participant 005)

“*I never saw a reason to ask my mother… I trust that my mother will not give me something which will harm me.*”

(Participant 007)

“*I feel fine, I trust my father, I don’t believe he can give me something that kills.*”

(Participant 016)

“*My treatment is fine; I don’t have side effects… I feel like I am in jail because I must mind time always.*”

(Participant 012)

“*I get dizzy after taking my pills.*”

(Participant 005)

“*Whenever I take my medication, I feel sick… I think it is because of the ARVs.*”

(Participant 011)

“*Sometimes I get tired of taking treatment… The thought of taking treatment daily for the rest of my life sometimes scares me.*”

(Participant 011)

“*Our lives are in danger as we have to take treatment every day… failing to do that could mean the end of our lives.*”

(Participant 002)

#### 3.1.3. Sub-Theme 1.3: Knowledge Related to Contraindications of Mixing Herbs and Pills

Adolescents reported knowing that mixing traditional medicine with ARVs is dangerous. None reported using herbs together with treatment.

“*Yes, nurses told me that if I mix traditional medicine with my pills, I will get sick…*”

(Participant 001)

“*I know that I must not mix my medication with any medication that is not from the clinic or the doctor.*”

(Participant 004)

#### 3.1.4. Sub-Theme 1.4: HIV-Positive Status Disclosure to Adolescents

Less than half of the adolescents received formal disclosure from parents, guardians, or professionals. Others discovered their status on their own or were given alternative explanations for the medication.

“*In 2017 when I fell pregnant, the nurses told me that I was born with HIV…*”

(Participant 015)

“*I asked my sister why I am taking this medication, then she explained to me that I was born with HIV…*”

(Participant 006)

“*No one told me, I have just discovered it along the way… I do a lot of reading about HIV.*”

(Participant 002)

“*I was never told what the medication is for but when I read my file it’s written RVD.*”

(Participant 005)

“*My father told me that the medication that I am taking were for the tonsils.*”

(Participant 013)

“*I was told by my mother that I am living with HIV… it doesn’t mean that I am different from other people.*”

(Participant 011)

“*Two years back, my mother told me… that I am living with HIV.*”

(Participant 012)

#### 3.1.5. Sub-Theme 1.5: Knowledge of the Dangers of Unprotected Sexual Intercourse

Adolescents demonstrated understanding of the risks associated with unprotected sexual intercourse, including HIV transmission and unintended pregnancy.

“*Yes, but I intend to use condoms every time I have sex…*”

(Participant 002)

“*Yes, they protect us from unwanted pregnancies and sexually transmitted diseases like HIV/AIDS.*”

(Participant 004)

“*If I had unprotected sexual intercourse, I could pass the virus to my partner… So, I have decided to wait.*”

(Participant 002)

### 3.2. Theme 2: Support Systems

#### 3.2.1. Sub-Theme 2.1: Support from Clinic Nurses

A quarter of adolescents reported receiving emotional support from nurses. Many said nurses treated them well, offered encouragement, and provided advice on medication adherence.

“*Nurses are treating me well… they advise me not to give up.*”

(Participant 017)

“*I feel at home when I am at the clinic…*”

(Participant 001)

However, some adolescents reported not receiving adequate information regarding their diagnosis or medication and felt unable to ask questions.

“*I have questions… but I am afraid to ask.*”

(Participant 010)

“*No, nurses don’t teach me anything related to my disease.*”

(Participant 010)

#### 3.2.2. Sub-Theme 2.2: Parental and Community Support

Many adolescents adhered to medication because of strong parental support. Others, especially those who had lost parents, described mixed experiences; some received practical and emotional support from relatives, while others experienced negative or emotionally harmful interactions.

“*My sister supports me emotionally and financially…*”

(Participant 008)

“*My granny… prepares food for me before taking my medication.*”

(Participant 010)

“*My aunt would say I was not sick… Sometimes she would tell her friends…*”

(Participant 013)

“*I feel angry like I am a real orphan.*”

(Participant 007)

“*Sometimes I cry… When I think about my mom…*”

(Participant 001)

#### 3.2.3. Sub-Theme 2.3: Support at School from Friends and Teachers

Adolescents generally reported good relationships with peers and teachers, but they did not receive HIV-related support because they had not disclosed their status due to fear of stigma. Some participants who believed their friends would not treat them badly, but still felt uncomfortable disclosing

“*I have a good relationship with everyone at school.*”

(Participant 010)

“*They would treat me differently…*”

(Participant 010)

“*They will play with me well… but I would not feel well.*”

(Participant 005)

“*My best friend knows that I have to take my pills every day…*”

(Participant 015)

“*My friend knows that I collect treatment monthly…*”

(Participant 002)

“*I feel bad, I don’t accept it…*”

(Participant 014)

## 4. Discussion

This study explored and identified challenges experienced by adolescents living with perinatally acquired HIV to develop a support programme for them. In interpreting these findings, it is important to recall the two-step analytical approach: the inductive themes of HIV/AIDS knowledge and support systems emerged directly from participant narratives, while the application of Life Course Theory provided a subsequent interpretive lens for understanding the developmental and social mechanisms underlying these experiences. The findings of this study reported that most adolescents have basic knowledge of HIV transmission and prevention, especially in older adolescents and those with the highest level of education. In agreement with the findings of the present study, previous studies conducted in sub-Saharan Africa reported similar findings, demonstrating that increases in HIV awareness among adolescents living with HIV are related to age and education [23,24].

While the adolescents were generally aware of how HIV is transmitted and prevented, more than half of the participants (54.8%) reported a desire for more information about HIV/AIDS, particularly about prevention techniques and the adverse effects of antiretroviral treatment. These findings are consistent with studies from Kenya and Uganda, which found that adolescents relied heavily on parents or caregivers for medication adherence and had low HIV knowledge [25,26].

The patterns of delayed, informal, or contextual disclosure that were noted are in line with previous research. Studies from South Africa and other low- to middle-income countries show that caregivers often delay disclosure due to fears of psychological harm or stigma, leading to adolescents discovering their status accidentally or later in life [27,28]. Similar to our findings, inadequate post-disclosure counselling has been linked to confusion, emotional distress, and difficulty coping with the diagnosis [29,30]. While early, structured disclosure has been associated with better adherence and psychological adjustment, most adolescents in this study lacked ongoing support afterward, possibly explaining their ongoing emotional struggles [31]. This gap highlights the disparity between WHO guidelines and actual practice [32].

In terms of emotional well-being, the quantitative data revealed that a considerable proportion of adolescents experienced significant psychological distress, with 32.9% reporting feeling guilty about their HIV status and 26.3% sometimes feeling useless or wishing they were dead. The qualitative findings provide a deeper, contextual understanding of these statistics. For instance, the quantitative finding of guilt is vividly illustrated by the qualitative accounts of stigma and family strain. An adolescent’s statement, “*My aunt would say I was not sick… Sometimes she would tell her friends…*” (Participant 013), sheds light on how discriminatory family environments can foster feelings of shame and guilt, which are then captured in the survey data. Furthermore, the feeling of being “useless” or wishing for death, reported by over a quarter of respondents, is given a human voice by the orphanhood and loss expressed qualitatively: “*I feel angry like I am a real orphan*” (Participant 007) and “*Sometimes I cry… When I think about my mom…*” (Participant 001). This weaving of data types shows that the quantitative prevalence of distress is not just a number, but a reflection of profound, lived experiences of loss, stigma, and fractured support systems. Conversely, the resilience noted in the quantitative data (65.3% not feeling helpless) is echoed in the qualitative resolve of participants like Participant 002, who stated, “*I intend to use condoms… I have decided to wait*,” demonstrating agency despite their circumstances. These findings are consistent with prior research showing elevated emotional distress among ALWH [33,34]. Notably, over a quarter of them sometimes felt useless or wished they were dead, reflecting increased vulnerability to depression compared to HIV-negative peers. Despite this, most adolescents did not feel helpless about their status, indicating resilience. This contrasts with studies reporting pervasive hopelessness, especially in contexts of poverty or limited healthcare, suggesting a complex coexistence of resilience and distress influenced by family support, healthcare relationships, and disclosure experiences [33,34].

Family support was identified as a key factor influencing coping and treatment adherence, aligning with research across Africa showing that supportive caregiving improves emotional well-being among ALWH [35,36]. Those reporting supportive family relationships shared these positive outcomes. However, adolescents who experienced parental loss due to HIV displayed poorer psychosocial outcomes, including feelings of exclusion and emotional neglect [37]. Unlike some studies that report overt household discrimination, most adolescents in this study experienced subtle, nuanced forms of family strain [38,39].

The role of nurses as both clinical and emotional support agents is well supported by existing research. Studies from high HIV-burden areas highlight nurses as trusted providers for adolescents with HIV [40,41]. However, consistent with our findings, many adolescents reported unmet needs for information, counselling, and meaningful engagement during clinic visits. The desire for nurses to spend more time discussing HIV status and treatment reflects the broader call for adolescent-friendly services. The limited use of support groups here suggests underutilization of potentially protective interventions, unlike settings where peer counsellors and dedicated adolescent clinics are more common. Adolescents generally maintained positive peer and teacher relationships but avoided disclosing their status due to fear of differential treatment, similar to prior studies [42,43]. While some research describes enacted stigma in schools, most adolescents here reported anticipated stigma, which aligns with findings that fear of disclosure often exceeds actual discrimination [44,45].

Interpreting the findings through the Life Course Theory framework provides insight into the developmental and social factors shaping adolescents’ experiences with HIV. The construct of timing is reflected in adolescents’ varied experiences of HIV status disclosure, while linked lives highlights the role of family members, caregivers, and healthcare providers in shaping coping and treatment adherence. The construct of agency is evident in adolescents’ efforts to manage their condition and navigate social relationships following disclosure. Although this study was initially conceptualized to inform the development of a support programme for adolescents living with perinatally acquired HIV, the present paper reports the needs assessment phase. The findings reveal important gaps in HIV knowledge, disclosure processes, emotional well-being, and supportive relationships, providing evidence to inform the development of a context-specific support programme grounded in Life Course Theory.

## 5. Conclusions

This study provides important insights into the informational, psychosocial, and support needs of adolescents living with perinatally acquired HIV in rural Limpopo. The findings highlight key challenges related to HIV knowledge, disclosure experiences, emotional well-being, and the availability of supportive relationships. These results provide a foundation for the future development of a Life Course-informed support programme that addresses the developmental and social realities of adolescents living with perinatally acquired HIV. Future research should translate these findings into structured interventions aimed at strengthening psychosocial support, improving treatment adherence, and enhancing the overall well-being of adolescents living with HIV. While efforts were made to confirm perinatal HIV acquisition through clinical records, caregiver reports, and consultation with healthcare providers, the possibility of misclassification cannot be entirely excluded. In the absence of definitive early infant diagnosis records for all participants, this study relied on documented treatment history since early childhood and ART initiation before age 10 as proxy indicators. Future research should consider incorporating biomarker confirmation or a comprehensive longitudinal clinical record review to strengthen the certainty of perinatal HIV classification.

## Figures and Tables

**Table 1:** Characteristics of participants identified with perinatally acquired HIV (N = 186).

Variables	Category	Frequency (n)	Percentage (%)
**Age**	12–15 years	91	48.9
	16–19 years	95	51.1
**Gender**	Female	100	53.8
	Male	86	46.2
**Education**	Primary	64	34.4
	Secondary	116	62.4
	Tertiary	6	3.2
**Residence type**	Rural	106	57.0
	Semi-rural	62	33.3
	Urban	18	9.7
**Staying with parents**	Yes	104	56.5
	No	82	43.5
**Lost a parent due to HIV**	Yes	54	29.0
	No	132	71.0

**Table 2:** Adolescents’ emotional assessment (n = 170).

Emotional Aspect	Yes n (%)	No n (%)
**Have been admitted or bedridden due to HIV**	26 (15.3)	144 (84.7)
**Have ever received help regarding your problem**	57 (33.5)	113 (66.5)
**HIV status caused problems with family members**	29 (17.1)	141 (82.9)
**Lost friends because of your condition**	30 (17.6)	140 (82.4)
**HIV condition disturbed school activities**	40 (23.5)	130 (76.5)
**Feel guilty about HIV status**	56 (32.9)	114 (67.1)
**Lost interest in activities previously enjoyed**	30 (17.6)	140 (82.4)
**Attended support group for adolescents living with HIV**	38 (22.4)	132 (77.6)

Note: Sample size reflects 170 adolescents who provided complete responses to all emotional assessment items. Percentages are calculated based on this valid response sample (n = 170). Sixteen participants (8.6% of the total sample) had missing data for one or more emotional items and were excluded listwise from this analysis.

**Table 3:** Assessment of adolescents’ coping status, n = 186% in rows: n (%).

Statements	Agree n (%)	Neutral n (%)	Disagree n (%)
**Need more knowledge regarding HIV/AIDS**	102 (54.8)	66 (35.5)	18 (9.7)
**Feel helpless about my HIV positive status**	22 (12.9)	37 (21.8)	111 (65.3)
**Feel like I am not coping with taking medication every day**	66 (35.5)	80 (43.0)	40 (21.5)
**Don’t know how to get out of this situation**	56 (30.1)	75 (40.3)	55 (29.6)
**The health professional attends to me when I come for a consultation**	74 (39.8)	53 (28.5)	59 (31.7)
**Wish nurses can also give attention when I come for my consultation and medication**	100 (53.8)	43 (23.1)	43 (23.1)
**Wish the nurses can talk to me about my HIV status when I come for my consultation**	95 (51.1)	46 (24.7)	45 (24.2)
**Feel sometimes I need clinical counselling regarding my condition**	72 (38.7)	57 (30.6)	57 (30.6)
**Family is supportive of my situation**	83 (44.6)	54 (29.0)	49 (26.3)
**Feel like I am losing control of my situation**	71 (38.2)	66 (35.5)	49 (26.3)
**Have fear about my future**	68 (36.6)	55 (29.6)	49 (26.3)
**My lifestyle has changed since I started taking my ARVs**	37 (19.9)	49 (26.3)	100 (53.8)
**Family do not treat me like other children at home**	53 (28.5)	51 (27.4)	82 (44.1)
**Sometimes feel useless and wish I was dead**	49 (26.3)	45 (24.2)	92 (49.5)

**Table 4:** Themes, Sub-themes, and Life Course Theory Interpretation.

Inductive	Inductive Sub-Theme	Verbatim Quote (Participant ID)	Life Course Theory Construct	Theoretical Interpretation
**HIV/AIDS Knowledge**	Knowledge of HIV/AIDS and self-HIV-positive status	“HIV is a disease that is caused by mixing of two types of blood where one person is infected by HI virus… It can also be transmitted from the mother to an unborn child.” (Participant 002)	Timing in lives	Knowledge acquisition is age-graded; older adolescents with more education demonstrate more sophisticated understanding, reflecting how developmental timing shapes health literacy.
	Knowledge of ARVs and related side effects	“I don’t know what the treatment is for… I must take them every day, or I will die.” (Participant 016)	Human agency	Limited treatment literacy constrains adolescents’ ability to exercise agency over their health, reducing adherence from informed choice to fearful compliance.
	Knowledge of contra-indications of mixing herbs with pills	“Yes, nurses told me that if I mix traditional medicine with my pills, I will get sick…” (Participant 001)	Linked lives	Health knowledge is transmitted through social connections with healthcare providers, demonstrating how linked lives influence health behaviours.
	HIV-positive status disclosure to adolescents	“No one told me, I have just discovered it along the way… I do a lot of reading about HIV.” (Participant 002)	Timing in lives	Delayed or absent disclosure represents a critical juncture where the timing of life events (learning one’s status) shapes subsequent health trajectories.
	Knowledge of the dangers of unprotected sexual intercourse	“If I had unprotected sexual intercourse, I could pass the virus to my partner… So, I have decided to wait.” (Participant 002)	Human agency	Despite challenges, adolescents demonstrate agency by making conscious decisions to protect others, reflecting forward-thinking life planning.
**Support Systems**	Support from clinic nurses	“I have questions… but I am afraid to ask.” (Participant 010)	Linked lives	Healthcare relationships are pivotal social connections that can either facilitate or hinder adolescents’ ability to manage their condition.
	Parental and community support	“My aunt would say I was not sick… Sometimes she would tell her friends…” (Participant 013)	Linked lives	Family relationships shape developmental trajectories; negative family interactions can become sources of stress rather than support.
	Support at school from friends and teachers	“They will play with me well… but I would not feel well.” (Participant 005)	Life-span development	Social relationships across different contexts (school, community) influence psychosocial development and identity formation during adolescence.

## Data Availability

The data presented in this study are available upon request from the corresponding author. The data are not publicly available due to privacy or ethical restrictions.

## Supplementary Materials

The following supporting information can be downloaded at: https://www.mdpi.com/article/10.3390/ijerph23040441/s1, Table S1: Integration of Qualitative and Quantitative Findings: Meta-Inferences on HIV/AIDS Knowledge, Disclosure, Emotional Well-being, Resilience, and Healthcare Support.
