# Supplementary material for: Needs Assessment to Inform a Life Course-Based Support Programme for Adolescents with Perinatally Acquired HIV in Rural Limpopo, South Africa
Source: Int J Environ Res Public Health. 2026 Mar 31;23(4):441. doi: 10.3390/ijerph23040441 (PMC13116131; doi:10.3390/ijerph23040441)
Supplement: Supplementary file 1 [file ijerph-23-00441-s001.zip › ijerph-4166013-supplementary.pdf]

Table S1: Integration of Qualitative and Quantitative Findings: Meta-Inferences on HIV/AIDS Knowledge, Disclosure, Emotional Well-being, Resilience, and Healthcare Support

Qualitative Theme	Illustrative Quote	Quantitative Item	Key Quantitative Finding	Meta-Inference (Integrated Finding)
**HIV/AIDS Knowledge**	*"I don't know what the treatment is for... I must take them every day, or I will die."* (Participant 016)	Need more knowledge regarding HIV/AIDS	54.8% agreed they need more knowledge.	Despite daily ART, a significant knowledge deficit exists, driven by incomplete or fear-based disclosure. Knowledge is often procedural ("take pills") rather than conceptual ("why the pills work").
**Disclosure**	*"No one told me, I have just discovered it along the way... I do a lot of reading."* (Participant 002)	N/A (qualitative finding only)	N/A.	Accidental or self-discovery of status is a common and distressing experience, highlighting a major gap in adherence to disclosure guidelines and a critical need for structured, supported disclosure processes.
**Emotional Well-being**	*"My aunt would say I was not sick... Sometimes she would tell her friends..."* (Participant 013)	Feel guilty about your HIV positive status	32.9% reported feeling guilty.	The quantitative prevalence of guilt is directly linked to qualitative accounts of stigma and unsupportive family environments. Emotional distress is not merely internal but is often a product of negative social interactions.

**Resilience and Agency**	*"If I had unprotected sexual intercourse, I could pass the virus to my partner... So, I have decided to wait."* (Participant 002)	Feel helpless about my HIV positive status	65.3% disagreed that they feel helpless.	The majority of adolescents do not feel helpless, demonstrating significant resilience. This is reflected in their ability to articulate plans for preventing transmission, showing a sense of agency and responsibility for others.
**Healthcare Support**	*"I have questions... but I am afraid to ask."* (Participant 010)	Wish nurses can also give attention when I come for my consultation	53.8% wished for more attention from nurses.	A large gap exists between the clinical care provided and the adolescents' need for psychosocial support and open communication. The clinic setting, while physically accessible, is not yet perceived as a safe and engaging space for addressing emotional and informational needs.
